# Discrepancies in the Time Course of Sleep Stage Dynamics, Electroencephalographic Activity and Heart Rate Variability Over Sleep Cycles in the Adaptation Night in Healthy Young Adults

**DOI:** 10.3389/fphys.2021.623401

**Published:** 2021-03-24

**Authors:** Ai Shirota, Mayo Kamimura, Akifumi Kishi, Hiroyoshi Adachi, Masako Taniike, Takafumi Kato

**Affiliations:** ^1^Department of Oral Physiology, Osaka University Graduate School of Dentistry, Suita, Japan; ^2^Graduate School of Education, The University of Tokyo, Bunkyo-ku, Japan; ^3^Osaka University Hospital, Sleep Medicine Center, Suita, Japan; ^4^Osaka University Health and Counseling Center, Toyonaka, Japan; ^5^Department of Child Development, Osaka University United Graduate School of Child Development, Suita, Japan

**Keywords:** first-night effect, adaptation, sleep-stage transition, sleep-stage continuity, sleep cycle, EEG power, heart rate variability, sleep process

## Abstract

**Objective:**

The aim of the present study was to characterize the cyclic sleep processes of sleep-stage dynamics, cortical activity, and heart rate variability during sleep in the adaptation night in healthy young adults.

**Methods:**

Seventy-four healthy adults participated in polysomnographic recordings on two consecutive nights. Conventional sleep variables were assessed according to standard criteria. Sleep-stage continuity and dynamics were evaluated by sleep runs and transitions, respectively. These variables were compared between the two nights. Electroencephalographic and cardiac activities were subjected to frequency domain analyses. Cycle-by-cycle analysis was performed for the above variables in 34 subjects with four sleep cycles and compared between the two nights.

**Results:**

Conventional sleep variables reflected lower sleep quality in the adaptation night than in the experimental night. Bouts of stage N1 and stage N2 were shorter, and bouts of stage Wake were longer in the adaptation night than in the experimental night, but there was no difference in stage N3 or stage REM. The normalized transition probability from stage N2 to stage N1 was higher and that from stage N2 to N3 was lower in the adaptation night, whereas that from stage N3 to other stages did not differ between the nights. Cycle-by-cycle analysis revealed that sleep-stage distribution and cortical beta EEG power differed between the two nights in the first sleep cycle. However, the HF amplitude of the heart rate variability was lower over the four sleep cycles in the adaptation night than in the experimental night.

**Conclusion:**

The results suggest the distinct vulnerability of the autonomic adaptation processes within the central nervous system in young healthy subjects while sleeping in a sleep laboratory for the first time.

## Introduction

The sleep architecture throughout the night is continuous, but heterogeneous, and characterized by cyclic fluctuations. Alternating non-rapid eye movement (NREM) sleep and rapid eye movement (REM) sleep constitute a series of sleep cycles, with the latter being related to the sleep cycle configuration (Vyazovskiy and Delogu, 2014). Sleep cycles last for approximately 90 min (Hartmann, 1968) and are repeated 3–5 times in one night (Hartmann, 1968; Hirshkowitz et al., 1992; Rosipal et al., 2013). Mutual interactions between the genesis of NREM and REM sleep underlie the stability of sleep cycles overnight (Kishi et al., 2011; Hayashi et al., 2015). Sleep processes and continuity within one sleep cycle are characterized by dynamic phenomena such as transitions among sleep stages (Lo et al., 2004; Kishi et al., 2008, 2011). Sleep stages are associated with cortical electroencephalography (EEG) and autonomic nervous system activities (Žemaitytė et al., 1984; Toscani et al., 1996; Brandenberger et al., 2001); slow-wave sleep is characterized by high cortical delta power, whereas light NREM sleep and REM sleep are characterized by low delta power (Brandenberger et al., 2001). Reciprocal changes in sympathetic and parasympathetic modulation tone are correlated with the cortical delta power within a sleep cycle (Brandenberger et al., 2001). The ratio of sleep stages and activity levels of cortical and autonomic systems in a sleep cycle gradually change from the initial to late periods of sleep cycles (Dement and Wolpert, 1958; Feinberg, 1974; Brandenberger et al., 2001; Versace et al., 2003). Therefore, the time-course changes in sleep variables over sleep cycles represent the progression of sleep processes in the integration of cortical and autonomic homeostatic sleep regulation overnight (Hayashi et al., 2015).

Previous studies demonstrated that the progression and stability of sleep can be altered when one sleeps in an unfamiliar environment. A typical example is the first-night effect, a phenomenon that is commonly observed in the first night of polysomnographic recordings for the purpose of adaptation to sleep laboratory settings (Agnew et al., 1966). Sleep in the adaptation night is characterized by poorer sleep quality than in subsequent nights due to increased sleep latency, less REM sleep, and frequent arousal (Hirshkowitz et al., 1992; Sforza et al., 2008; Rosipal et al., 2013). A difference in the sleep architecture overnight between the adaptation and experimental nights was generally detected during the initial period of sleep or in the first sleep cycle such as a delay in the onset of NREM sleep stages and REM sleep latency (Agnew et al., 1966; Tamaki et al., 2005a). We therefore hypothesized that the sleep architecture in the first sleep cycle is most influenced by the laboratory environment in the adaptation night and the influences on sleep decrease in the subsequent sleep cycles in healthy subjects.

The characteristics of sleep in the adaptation night were previously investigated by conventional analyses of sleep architecture such as the amount of each sleep stage. Recent studies analyzed sleep continuity and characterized the patterns of sleep-stage transitions in order to elucidate the dynamic nature of sleep regulation (Kishi et al., 2017, 2020). The analyses in these studies revealed the novel properties of sleep regulation that were not detected by the conventional sleep-stage variables (Norman et al., 2006; Kishi et al., 2011). Therefore, the quantification of sleep continuity and sleep-stage transitions will enable the further characterization of sleep in the adaptation night. In addition, sleep variables assessed by sleep-stage scoring may not be concordant with those by the quantitative analyses of EEG and heart rate variability (HRV) activities in the adaptation night (Toussaint et al., 1997; Le Bon et al., 2001; Curcio et al., 2004; Israel et al., 2012; Virtanen et al., 2018). As such, analysis of sleep-stage dynamics and the quantification of cortical/cardiac activities may provide physiological insights into sleep processes over sleep cycles in the adaptation night. Therefore, the aim of the present study was to investigate the time-course changes in sleep-stage transitions, cortical EEG power, and heart rate variability in the progress of sleep cycles in the adaptation night in comparison with the experimental night in healthy subjects.

## Materials and Methods

### Participants

One hundred participants aged between 20 and 33 years (47 women and 53 men, mean age 24.3 ± 2.9 years) were enrolled in the PSG study at Osaka University between November 25th, 2013, and September 25th, 2019. The participants were recruited via flyers posted and word of mouth. They were compensated for participation (5,000 JPY). The sample size was determined by a prior power analysis in order to detect a medium effect size (dz = 0.5) with a power of 0.90 by a two-tailed paired *t*-test. All participants completed a written informed consent form approved by the Research Ethics Committee of Osaka University Graduate School of Dentistry and Osaka University Dental Hospital. This study was approved by the ethics committee of the Osaka University Dental Hospital and the Graduate School of Dentistry (H25-E9-5, H29-E48-3).

### Polysomnography and Sleep Stages

Polysomnographic recordings were performed on two consecutive nights in a sleep laboratory at Osaka University Graduate School of Dentistry. All subjects completed the Pittsburgh Sleep Quality Index (PSQI) for Japanese (Doi et al., 2000) and Self-rating Depression Scale: SDS (Zung, 1965): the Japanese version of the SDS (Fukuda and Kobayashi, 1973). The PSQI is a subjective questionnaire to assess sleep quality and disturbances over a 1 month period, and the SDS is a self-administered survey to quantify the depressed status of a patient. Subjects were instructed to lead a regular life prior to participating in the recording evaluation. They were not allowed to nap, perform excessive exercise, or drink alcohol before coming to the sleep lab on the two nights. On the day of the PSG recording, participants arrived at our sleep lab at approximately 8:30 pm. The light was off between 10:30 and 11:00 p.m. and on in the next morning between 6:30 and 7:30 a.m. or when they woke up. The times for lights-on and -off were the same between the two nights. After waking up, participants answered the questionnaire on the quality of sleep; they were asked about sleep latency, number of periods of wakefulness after sleep onset, total sleep time and, sleep quality score (from 1 point: “bad” to 5 points: “good”).

PSG recordings were performed using surface electroencephalography (EEGs: C3-A2, C4-A1, O1-A2, O2-A1, F3-A2, F4-A1, Fp1-A2, and Fp2-A1), bilateral electrooculography (EOG), lead II electrocardiography (ECG), and chin electromyography (EMG). Signals were amplified, filtered (EEG, EOG, and ECG: 0.3–70 Hz; EMG: > 10 Hz, with a 60 Hz hum filter), and recorded with a sampling frequency of 200 Hz using a software package (Embla N7000, REMbrandt^TM^ PSG software, Natus Medical, Pleasanton, CA). The stage was scored by one technician (registered polysomnographic technologist) blinded to the study aims (Nonoue et al., 2017). Oronasal thermal airflow, nasal pressure, chest, and abdominal movements, arterial oxygen saturation, and body position were also recorded. Audio and video recordings were performed simultaneously. Sleep stages and respiratory events were scored according to the American Academy of Sleep Medicine criteria version 2.1 (Berry et al., 2014). The apnea–hypopnea index (AHI) was calculated as the sum of all apneas and hypopneas with 3% O_2_ desaturation and/or EEG arousal divided by the total sleep time. The AHI exclusion criteria followed the AASM criteria (Berry et al., 2014). Subjects with AHI ≥5 times/h were excluded from the analyses of this study; they may exhibit a lower quality of sleep than the others even though they did not exhibit signs or symptoms of sleep apnea (Okura et al., 2020).

### Sleep Cycles

The sleep cycle was assessed with reference to the method proposed by Feinberg (1974). A sleep cycle was defined as the time from the end of REM sleep to the end of the next REM sleep. The first sleep cycle was defined as the time from sleep onset to the end of the first REM sleep. REM sleep was considered to be two separate REM sleep periods if it discontinued for more than 20 min and one REM sleep period if the gap was less than 20 min. Furthermore, participants were considered to have been in a REM sleep period if they woke up less than 10 min from the last REM sleep even if the sleep stage immediately before the end of the PSG recording was not REM sleep. Sleep cycles were considered to have been completed only if another sleep stage was continued more than 10 min from the last REM sleep and the number of complete cycles was measured accordingly. The number of REM sleep periods included those in which the last stage of the sleep cycle before waking up was REM sleep. In order to compare the variables (i.e., sleep stage, cortical activity, and heart rate variability) for sleep cycles between the adaptation night and the experimental night within a subject, the same number of sleep cycles was analyzed because the number of sleep cycles differed between the two nights in some subjects.

### Sleep-Stage Transition

The number of sleep-stage transitions per night was measured for each participant, and the rate of stage transitions (%) was calculated by dividing the number of sleep transitions per night by the total number of epochs. The same method was used to calculate the percentage of sleep stages for each sleep cycle. Normalized transition probabilities between five sleep stages were calculated by dividing the number of transitions from the specific stage to one of the other stages by the total number of transitions from the specific stage to another stage (Kishi et al., 2008, 2011).

The continuity of sleep (regardless of sleep stage) and each sleep stage (N1, N2, N3, REM, and wake) were analyzed with the rules in the previous study (Kishi et al., 2017). A sleep run began with a transition from Wake to any stage of sleep and continued until Wake occurred. Separate from the sleep run, a run of each sleep stage (N1, N2, N3, REM, and Wake) was defined as consecutive epochs scored as the stage, terminated by one or more epochs scored as another stage.

### Cortical and Cardiac Activities

During the night, continuous EEG (C4 referenced to the left ear) was digitized at 200 Hz and stored for off-line analysis. Prior to the analysis, epochs with artifacts were visually identified and removed. Spectral power was calculated using the fast Fourier transform (FFT) algorithm (Bio Trend Professional, NoruPro Light Systems). FFT windows of 2,048 points were used, and truncating error was reduced by applying a Hanning window. The frequency resolution was 0.25 Hz. The analysis window was 10.24 s with a 0.24 s overlap every 10 s, and the data for three units were averaged to obtain a value every 30 s. The limits for band frequencies were as follows: delta, 0.5–4 Hz; theta, 4–8 Hz; alpha, 8–12 Hz; sigma, 12–15 Hz; low beta, 15–23 Hz; high beta, 23–32 Hz.

Heart rate analysis was performed using complex demodulation (CD) (Shin et al., 1989). The oscillations can be characterized based on the heart rate accelerating or slowing, the wavelength, and/or the amplitude (Task Force of the European Society of Cardiology and the North American Society of Pacing and Electrophysiology, 1996). The correlations of frequency components of HRV, however, diminish as the wavelength of the oscillations and the recording duration increase. The CD method uses the techniques of interpolation and detrending (Shin et al., 1989; Hayano et al., 1993) and provides the time resolution necessary to detect short-term heart rate changes and to describe the amplitude and phase of particular frequency components as functions of time (Task Force of the European Society of Cardiology and the North American Society of Pacing and Electrophysiology, 1996). The mean value of the instantaneous amplitude (in ms) was calculated in 30 s windows using a computer program with a time scale of 0.1 s and a frequency resolution of 0.002 Hz (HRV LOG-Pro-DSA Analysis, NoruPro Light Systems). Following the removal of epochs with artifacts, frequency spectra in RR interval data were estimated for the range between zero and 0.40 Hz and were divided into three components depending on their central frequencies, i.e., the spectral domain with a central frequency of less than 0.04 Hz, between 0.04 and 0.15 Hz, and greater than 0.15 Hz but less than 0.40 Hz. These domains were labeled as bands with a very low frequency (VLF), low frequency (LF), and high frequency (HF), respectively (Malliani et al., 1991). HRV reflects autonomic modulation, whereas the average RR interval reflects autonomic tone. In the present study, HF amplitude was used as an index of alteration of parasympathetic nervous system activity.

### Statistical Analysis

Paired *t*-tests were used to compare the adaptation and experimental nights, in addition to the mean duration of runs for sleep and each sleep stage. The effect size was presented in Cohen’s d, which was the mean preference index divided by the standard deviation. To individually assess variables that were significantly affected by the first-night effect, a three-way analysis of variance (ANOVA) for repeated measures [(night: two levels), (sleep stage: five levels), and (cycle: four levels)] was used. Moreover, a two-way analysis of variance (ANOVA) for repeated measures [(night: two levels) and (cycle: four levels)] was used to assess EEG and HRV parameters. The Greenhouse–Geisser ε correction was performed to evaluate *F*-ratios for repeated measures involving more than one degree of freedom and when the sphericity assumption was not met. The effect size was presented in partial η^2^, which was the sum of squares for the effect of interest divided by the total sum of squares for all data variance. *Post hoc* comparisons between pairs of nights were conducted using the paired *t*-test. Results were considered to be significant when *p*-values were less than 0.05.

## Results

### Participants

Data from 26 participants were excluded from the analysis for the following reasons: sleep apnea syndrome (AHI ≥ 5 for both nights) (*n* = 19) and TST/TIB < 70% or sleep latency > 60 min (*N* = 7). As a result, 74 participants were included (40 women and 34 men, 20–33 years old, mean age 23.8 ± 2.2 years, BMI 20.5 ± 1.6 kg/m^2^). The sleep quality assessed by the PSQI in all participants was 4.6 ± 1.96 (range: 0–21, cutoff score: 5.5≥). The average score on the SDS was within the standard range (range: 20–80, cutoff score: 40>, average score in all participants: 39.2 ± 5.49).

### Sleep Variables for the Entire Night

Sleep variables measured during the adaptation and experimental nights are shown in Table 1. Although the time in bed did not significantly differ between the two nights, the total sleep time was shorter (*p* < 0.01) and sleep efficiency was lower (*p* < 0.001) in the adaptation night than in the experimental night. The latencies of stage N1 (i.e., sleep latency), stage N3, and stage REM were significantly longer in the adaptation night than in the experimental night (all *p* < 0.05). In terms of sleep stages, the adaptation night was characterized by a longer time in WASO (wakefulness after sleep onset) and stage N1 (both *p* < 0.01), and a shorter time in stage REM (*p* < 0.001) than in the experimental night. The number of REM sleep periods increased from the adaptation night to the experimental night (*p* < 0.05).

**TABLE 1:** Sleep variables in adaptation and experimental nights.

	**Adaptation night**		**Experimental night**			
	**Mean**	***SD***	**Mean**	***SD***	**Cohen’s d**	***p***
**Sleep architecture**						
Time in bed (min)	466.9	34.6	468.3	33.8	0.04	0.646
Sleep efficiency (%)	91.6	4.9	94.1	4.0	0.57	<0.001**
**Latency (min)**						
Sleep latency	7.8	7.8	6.0	5.6	0.26	0.047*
N2 latency	11.5	8.4	9.9	7.3	0.21	0.098
N3 latency	22.3	18.1	17.3	9.3	0.35	0.011*
REM latency	110.4	43.0	97.3	42.0	0.31	0.028*
**Percentage of sleep stages (%)**						
N1	11.5	4.9	10.2	4.1	0.30	0.003**
N2	43.8	7.4	44.4	6.6	0.09	0.456
N3	21.9	7.4	22.6	6.6	0.10	0.289
REM	16.3	4.2	18.5	4.1	0.51	<0.001**
WASO	6.5	4.5	4.3	3.5	0.54	<0.001**
Sleep cycles						
No. of REM sleep period	3.9	0.8	4.3	0.8	0.33	0.014*
**Stage transitions (%)**						
Percentage of stage transitions	20.7	4.2	18.9	3.9	0.45	<0.001**
**Arousal events**						
Arousal index	12.9	5.7	9.8	4.5	0.60	<0.001**
Respiratory events						
AHI (no./h)	2.4	2.1	2.1	1.7	0.16	0.196
**Subjective sleep parameters**						
Sleep latency (min)	20.0	17.7	15.0	11.0	0.34	0.017*
No. of WASO	2.9	1.8	2.3	1.4	0.41	0.003**
Total sleep time (min)	415.7	71.9	434.0	41.3	0.31	0.024*
Sleep quality	2.5	0.9	2.9	1.0	0.36	0.002**

SD, standard deviation; WASO, wakefulness after sleep onset; REM, rapid eye movement; AHI, apnea hypopnea index. *p < 0.05, **p < 0.01 from the experimental night by a two-tailed paired t-test.

The percentage of transitions per night (%) was significantly higher in the adaptation night than in the experimental night (*p* < 0.001, Table 1). The arousal index was significantly higher in the adaptation night than in the experimental night (*p* < 0.001), but the number of awakenings did not significantly differ between the two nights. No significant difference was observed in AHI between the two nights. The subjective sleep parameters were better in the experimental night than in the adaptation night (No. of WASO, total sleep time, and sleep quality) (all *p* < 0.05, Table 1).

### Sleep-Stage Continuity and Sleep-Stage Transitions for the Entire Night

The mean continuity time for sleep and each sleep stage is shown in Table 2. The mean duration of sleep runs was significantly shorter in the adaptation night than in the experimental night (*p* < 0.01). Runs of stage N1 and stage N2 were shorter in the adaptation night than in the experimental night (both *p* < 0.01), but no significant difference was observed in stage N3 or stage REM. On the other hand, the runs of stage Wake were significantly longer in the adaptation night than in the experimental night (*p* < 0.01).

**TABLE 2:** Mean continuity time for sleep and each sleep stage.

	**Adaptation night**		**Experimental night**			
	**Mean**	***SD***	**Mean**	***SD***	**Cohen’s d**	***p***
Sleep runs (min)	19.1	9.1	22.8	11.1	0.36	0.001**
N1 runs (min)	0.9	0.2	1.0	0.2	0.45	0.007**
N2 runs (min)	3.4	1.1	3.9	1.1	0.42	<0.001**
N3 runs (min)	7.0	5.3	6.1	2.4	0.22	0.129
REM runs (min)	9.1	6.0	8.8	5.1	0.06	0.590
Wake runs (min)	1.1	0.7	0.9	0.4	0.40	0.004**

Values are means and standard deviations, **p < 0.01 from the experimental night by a two-tailed paired t-test.

Normalized transition probabilities among the five vigilance states (Wake, N1, N2, N3, and REM) for the two nights are shown in Table 3. The transition from Wake to N1 (Wake → N1) in the adaptation night was significantly higher, whereas that from Wake to N2 (Wake → N2) was significantly lower in the adaptation night than in the experimental night (*p* < 0.01). Normalized transition probabilities from N1 to REM (N1 → REM) significantly decreased in the adaptation night than in the (*p* < 0.01). Normalized transition probabilities from N2 to N1 (N2 → N1) were significantly higher in the adaptation night than in the experimental night (*p* < 0.01), whereas those from N2 to N3 (N2 → N3) were lower in the adaptation night than in the experimental night (*p* < 0.01). No inter-night differences were found in transitions from stage N3 to other stages. The frequency of transition from REM to N1 (REM → N1) was significantly lower in the adaptation night than in the experimental night (*p* < 0.05), whereas that from REM to N2 (REM → N2) was higher in the adaptation night than in the experimental night (*p* < 0.01).

**TABLE 3:** Normalized transition probabilities between five behavioral states within the sleep period time in the adaptation and experimental nights.

**Adaptation night**								**Experimental night**							
			**Followed stage**								**Followed stage**				
	**(%)**	**Wake**	**N1**	**N2**	**N3**	**REM**			**(%)**	**Wake**	**N1**	**N2**	**N3**	**REM**	
	Wake		76.6**	16.8**	1.9	4.7**	100%		Wake		71.1	21.1	1.3	6.5	100%
	N1	14.0		77.1	0.6	8.3	100%		N1	12.1		75.8	0.7	11.4	100%
**Previous stage**	N2	19.0	44.9**		27.7**	8.4	100%	**Previous stage**	N2	18.1	38.9		33.6	9.4	100%
	N3	18.6	9.8	71.2		0.4	100%		N3	16.6	9.0	73.9		0.5	100%
	REM	32.7	45.4*	21.7**	0.2		100%		REM	32.7	52.0	15.3	0.0		100%

Normalized transition probabilities between Wake, N1, N2, N3, and REM within the sleep period time in the adaptation and experimental nights. The labels in the rows denote the preceding states, and those in the columns denote the subsequent states of transitions. *p < 0.05, **p < 0.01 from the experimental night by a two-tailed paired t-test.

### Sleep Variables for Each Sleep Cycle

**FIGURE 1:**
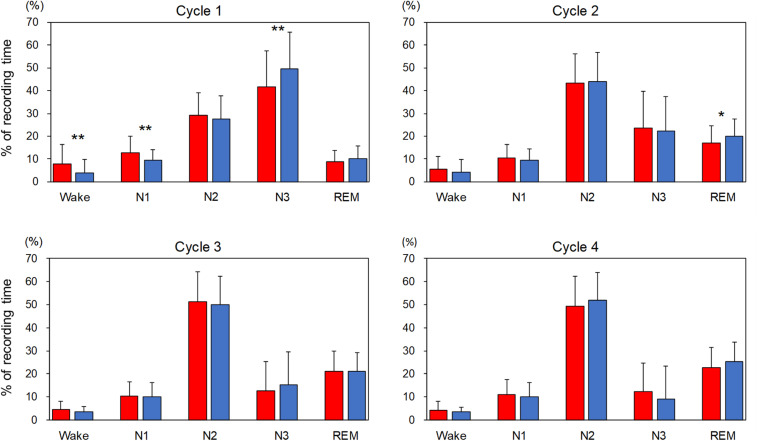
Percentage of each sleep stage in each sleep cycle. The percentage of each sleep stage is present as mean and standard deviation (red box: adaptation night, blue box: experimental night). Values that significantly differed between nights are indicated by one (*p* < 0.05) or two stars (*p* < 0.01; two-tailed paired *t*-test). The numbers of subjects were 74 for cycles 1 and 2, 63 for cycle 3 and 34 for cycle 4.

The percentage of each sleep stage in each sleep cycle is shown in Figure 1. In order to compare the variables for sleep cycles between the adaptation night and the experimental night within a subject, the same number of sleep cycles was analyzed (cycle 1 and cycle 2: *n* = 74, cycle 3: *n* = 63, cycle 4: *n* = 34). The three-way repeated-measures ANOVA (nights: two levels × sleep stage: five levels × sleep cycle: four levels) demonstrated a significant interaction [*F*_(__12_,_396__)_ = 2.82, *p* = 0.022, ε = 0.38, partial η^2^ = 0.085]. *Post hoc* comparisons between the two nights in the first sleep cycle revealed that the percentages of stage Wake and N1 were significantly higher (*p* < 0.01), and that of stage N3 was significantly lower (*p* < 0.01) in the adaptation night than in the experimental night. *Post hoc* comparisons between the two nights in the second sleep cycle revealed that the percentage of stage REM was significantly lower (*p* < 0.05) in the adaptation night than in the experimental night. There were no significant differences between the two nights in the third and fourth sleep cycles.

### Quantitative EEG and HRV Analyses

**FIGURE 2:**
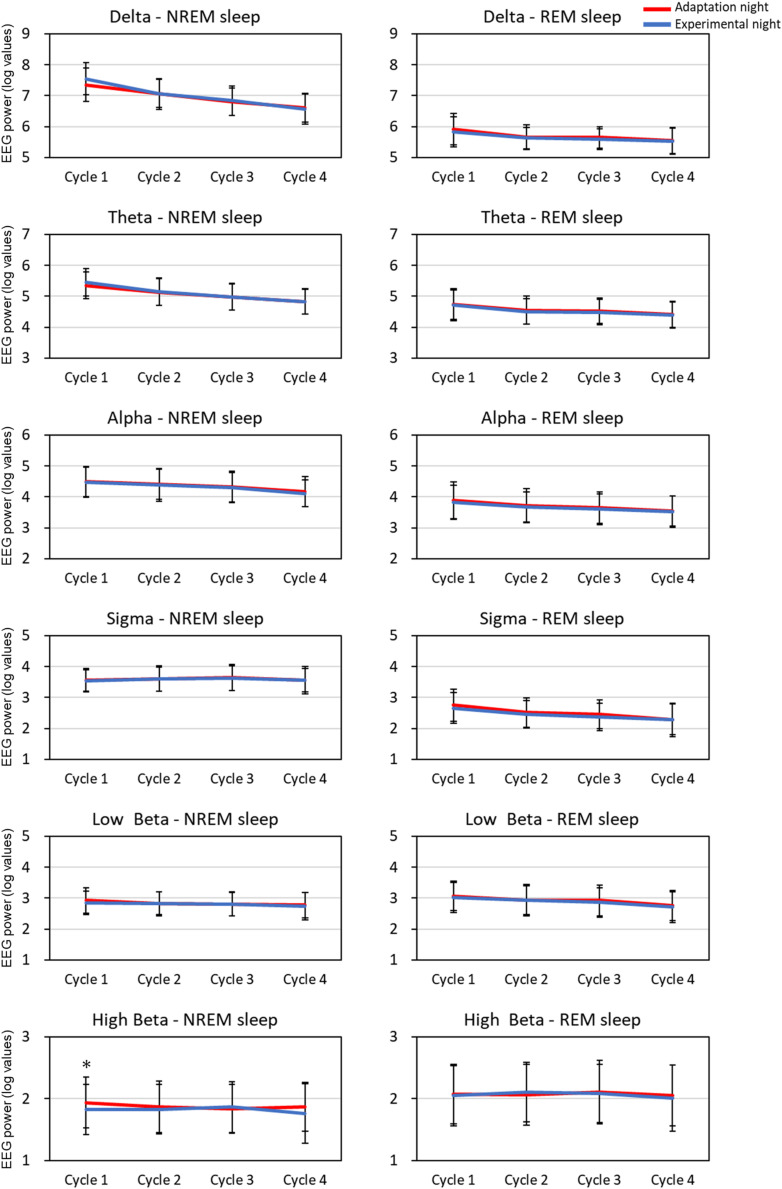
EEG spectral power for each sleep cycle. EEG power was expressed as log-transformed values. The data is present as mean and standard deviation (red line: adaptation night, blue line: experimental night). Values that significantly differed between nights are indicated by one star (*p* < 0.05; two-tailed paired *t*-test). The frequency ranges were as follows: delta, 0.5–4 Hz; theta, 4–8 Hz; alpha, 8–12 Hz; sigma, 12–15 Hz; low beta, 15–23 Hz; high beta, 23–32 Hz. The numbers of subjects analyzed were the same as in Figure 1.

The mean spectral parameters of the first four sleep cycles calculated on the adaptation night and experimental night are shown in Figure 2. The two-way repeated-measures ANOVA (nights: two levels × sleep cycle: four levels) demonstrated a significant interaction [*F*_(3,99)_ = 3.49, *p* = 0.019, partial η^2^ = 0.096] only in the high Beta band in the NREM sleep period. *Post hoc* comparisons between the two nights revealed that the high Beta band in NREM of the first sleep cycle was significantly higher (*p* < 0.01) in the adaptation night than in the experimental night. There were no significant differences between the two nights in all other bands in NREM and REM sleep periods.

**FIGURE 3:**
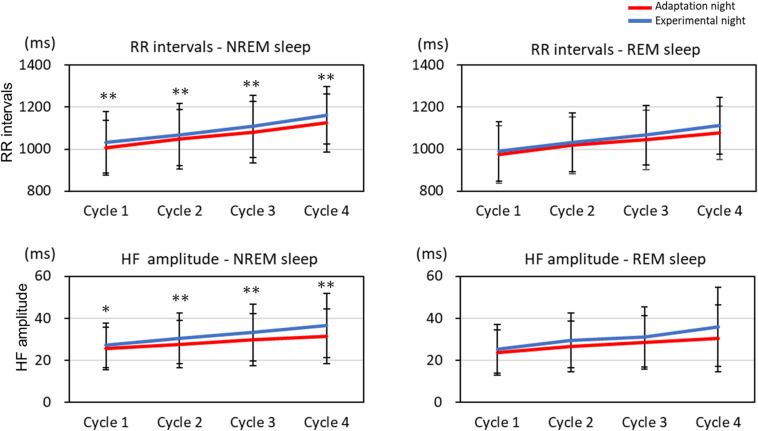
RR intervals and HF amplitude for each sleep cycle. RR intervals and HF amplitude for each sleep cycle are present as mean and standard deviation (red line: adaptation night, blue line: experimental night). Values that significantly differed between nights are indicated by one (*p* < 0.05) or two stars (*p* < 0.01; two-tailed paired *t*-test). The numbers of subjects analyzed were the same as in Figure 1.

The mean HF band and RR intervals of the first four sleep cycles calculated on the adaptation night and experimental night are shown in Figure 3. In the NREM sleep period, the two-way repeated-measures ANOVA (nights: two levels × sleep cycle: four levels) revealed a significant interaction between nights and sleep cycles for the RR intervals and HF amplitude [RR intervals: *F*_(3, 99)_ = 2.88, *p* = 0.040, partial η^2^ = 0.080; HF amplitude: *F*_(3, 99)_ = 3.08, *p* = 0.031, partial η^2^ = 0.085]. *Post hoc* comparisons between the two nights revealed that RR intervals and HF amplitude were significantly lower over the four sleep cycles in the adaptation night than in the experimental night (both *p* < 0.05). There were no significant differences between the two nights in RR intervals or HF amplitude in the REM sleep periods.

## Discussion

The present study investigated the process to adaptation to sleeping in a sleep laboratory in healthy young adults. The objective and subjective sleep quality was lower in the adaptation night than in the experimental night and was characterized by low sleep continuity and high sleep-stage transitions in association with the changes in cortical EEG power and heart rate variability. Cycle-by-cycle analyses revealed differences in sleep-stage distribution and cortical beta EEG power in the first sleep cycle. However, heart rate variability differed in the four sleep cycles between the two nights. This suggested that the physiological systems representing sleep-stage dynamics, cortical activity, and heart rate variability were differentially altered during the progression of sleep cycles between the adaptation and experimental nights in healthy subjects.

### Sleep-Stage Dynamics in the Entire Night and Sleep Cycles

The present study revealed that the sleep macrostructure on the adaptation night was characterized by reduced sleep efficiency, less REM sleep, more stage N1, more wakefulness, and more frequent arousal than in the experimental night (Table 1). This study supports previous findings on the so-called first-night effect (Agnew et al., 1966; Browman and Cartwright, 1980; Toussaint et al., 1995; Rotenberg et al., 1997; Le Bon et al., 2001; Curcio et al., 2004; Moser et al., 2010), although sleep architecture was of good quality for the two nights in this study population (e.g., sleep efficiency > 90%). In the adaptation night, the decreased stability of sleep stages can influence the sleep process. However, cycle-by-cycle analysis revealed that the first sleep cycle was most affected with higher percentage of time in stage N1 and stage Wake and lower percentage of stage N3 (Figure 1). Moreover, reduced sleep stability can disturb the progression of sleep stages in the first sleep cycle such as a delay in the onset of stage N3 and stage REM. However, in this study, the changes in sleep-stage dynamics in the adaptation night had no major impact on sleep architecture in the following sleep cycles. This suggests that the sleep in the first sleep cycle is susceptible to environmental changes in the adaptation night. However, environmental influences on sleep processes are not carried over to later sleep cycles because mutual interactions between homeostatic sleep regulation and ultradian rhythms may have functioned in young healthy participants in this study (Lorenzo and Barbanoj, 2002; Hayashi et al., 2015; Kishi et al., 2011, 2018).

### Quantitative EEG and HRV Variables

Cortical EEG power, such as delta bands, autonomic nervous system function, and sleep-stage distribution fluctuate within a sleep cycle (Brandenberger et al., 2001; Borbély et al., 2016; de Zambotti et al., 2018). Previous studies reported the difference in the sleep architecture between the adaptation (first) and experimental (second) nights in healthy subjects, but the difference in sleep architecture was not clearly correlated with that in EEG power spectra (Toussaint et al., 1997; Curcio et al., 2004) or HRV (Israel et al., 2012; Virtanen et al., 2018) throughout the entire night. Based on the cycle-by-cycle analyses of both nights, the cortical EEG power for each frequency band and HRV of NREM sleep and REM sleep exhibited typical alterations across sleep cycles such as the decrease in delta EEG power and increase in RR intervals (Achermann and Borbély, 2017; Lanfranchi et al., 2017; de Zambotti et al., 2018). However, the time-course changes in sleep architecture, EEG activity, and HRV can differ among the sleep cycles since cyclic fluctuation within a sleep cycle is modulated by the homeostatic and circadian influences over the night (Åkerstedt et al., 1998). As addressed above, in the first sleep cycle, sleep architecture differed between the adaptation and experimental nights. Delta EEG power did not differ between the two nights, as reported previously (Toussaint et al., 1997), whereas the beta EEG power was higher and RR intervals and HF amplitude were lower in the first sleep cycle in the adaptation night than in the experimental night. In the following sleep cycles, however, the difference in sleep architecture and EEG power between the two nights disappeared, whereas the RR intervals and HF amplitude, as demonstrated previously (Virtanen et al., 2018), remained lower in the adaptation night than in the experimental night (Figure 2). Previous studies showed that the correlation between cortical and autonomic activities was found to be attenuated in the latter half of the night in healthy subjects (Thomas et al., 2014; Rothenberger et al., 2015). Therefore, the distinct time course of cortical and autonomic activity suggests that the homeostatic and circadian influences can differently modulate the reactivity of cortical and autonomic activity in the adaptation night. The results also suggest the possibilities that the autonomic nervous system has lower adaptability than cortical system.

### Physiological Significance of Sleep-Stage Dynamics and Cortical/Cardiac Activity

Previous studies proposed that changes in sleep in the adaptation night are related to alertness in order to ensure safety when sleeping in a new and potentially dangerous environment (Curcio et al., 2004; Tamaki et al., 2016; Tamaki and Sasaki, 2019). Therefore, alertness may enhance wake-promoting influences at the beginning of sleep and increase the latency of sleep onset and NREM sleep stages in the adaptation night (Tamaki et al., 2005a,b). High beta EEG power and low RR intervals and HF amplitude may be associated with hyperarousal and/or autonomic hyperactivation related to alertness, with an increase in phasic cortical events related to autonomic activation (i.e., arousal and EEG desynchronization) in the adaptation night (Trinder et al., 2003; Kato et al., 2004; de Zambotti et al., 2011; Silvani et al., 2015). These conditions were reported in patients with sleep disorders such as chronic pain (Lavigne et al., 2011) and insomnia (Bonnet and Arand, 1997; Jurysta et al., 2009). However, in the healthy subjects of the present study, high beta EEG power and low RR intervals and HF amplitude may have a role for sleep maintenance, rather than sleep disturbance. As sensory alertness remains functional during sleep (Oswald et al., 1960; Kato et al., 2004; Lavigne et al., 2004), sensory experience in a novel sleep environment may be processed, especially during the first sleep cycle, to ensure the safety of the sleep laboratory environment in healthy participants: autonomic activity remains functional in order to respond to the environment in subsequent cycles.

### Study Limitations

The major factors causing altered sleep in a sleep laboratory have changed over time due to improvements in the sleep laboratory environment. However, disturbances cannot be completely eliminated by improving the comfort of the surrounding settings (Lorenzo and Barbanoj, 2002; Moser et al., 2010). In addition, sleep irregularity in the previous week negatively correlates with sleep efficiency during the adaptation night (Lee et al., 2016). In the present study, the possibility that sleep conditions prior to PSG recordings affected sleep in the laboratory recordings cannot be excluded because sleep–wake patterns on previous days were not fully controlled or monitored. Another limitation is that not all subjects had four sleep cycles in the adaptation and experimental nights. The results of cycle-by-cycle analysis performed on a limited number of subjects with four sleep cycles may be interpreted as the responses to the environmental influences in subjects whose sleep is more stable and regular than in those with fewer sleep cycles. Furthermore, psychological predisposition may play a role in the lower ability to adapt to a sleep laboratory environment. As the participants in the present study did not have self-rated depression, psychological effects on adaptability to the sleep laboratory environment were considered minimal.

## Conclusion

The present study revealed that the time course of sleep-stage dynamics, electroencephalographic activity, and heart rate variability over sleep cycles are discrepant in the adaptation night in healthy young adults. The results suggest the distinct vulnerability of the adaptation processes within the central nervous system while sleeping in a sleep laboratory for the first time.

## Data Availability Statement

The original contributions presented in the study are included in the article/supplementary material, further inquiries can be directed to the corresponding author/s.

## Ethics Statement

The studies involving human participants were reviewed and approved by the Research Ethics Committee of Osaka University Graduate School of Dentistry and Osaka University Dental Hospital (H25-E9-5, H29-E48-3). The patients/participants provided their written informed consent to participate in this study.

## Author Contributions

AS and TK designed the study and wrote the main manuscript. AS and MK prepared the data sets and analyzed the data. MK and TK contributed to the data collection. AK, HA, and MT revised and commented on the manuscript. All authors reviewed the manuscript and agreed with its content.

## Conflict of Interest

The authors declare that the research was conducted in the absence of any commercial or financial relationships that could be construed as a potential conflict of interest.
